# The Grass Might Be Greener: Medical Marijuana Patients Exhibit Altered Brain Activity and Improved Executive Function after 3 Months of Treatment

**DOI:** 10.3389/fphar.2017.00983

**Published:** 2018-01-17

**Authors:** Staci A. Gruber, Kelly A. Sagar, Mary K. Dahlgren, Atilla Gonenc, Rosemary T. Smith, Ashley M. Lambros, Korine B. Cabrera, Scott E. Lukas

**Affiliations:** ^1^Cognitive and Clinical Neuroimaging Core, McLean Imaging Center, McLean Hospital, Belmont, MA, United States; ^2^Marijuana Investigations for Neuroscientific Discovery Program, McLean Imaging Center, McLean Hospital, Belmont, MA, United States; ^3^Department of Psychiatry, Harvard Medical School, Boston, MA, United States; ^4^Department of Psychology, Tufts University, Medford, MA, United States; ^5^Behavioral Psychopharmacology Research Laboratory, McLean Imaging Center, McLean Hospital, Belmont, MA, United States

**Keywords:** medical marijuana, cannabis, neuroimaging, fMRI, cognition, executive function, MSIT

## Abstract

The vast majority of states have enacted full or partial medical marijuana (MMJ) programs, causing the number of patients seeking certification for MMJ use to increase dramatically in recent years. Despite increased use of MMJ across the nation, no studies thus far have examined the specific impact of MMJ on cognitive function and related brain activation. In the present study, MMJ patients seeking treatment for a variety of documented medical conditions were assessed prior to initiating MMJ treatment and after 3 months of treatment as part of a larger longitudinal study. In order to examine the effect of MMJ treatment on task-related brain activation, MMJ patients completed the Multi-Source Interference Test (MSIT) while undergoing functional magnetic resonance imaging (fMRI). We also collected data regarding conventional medication use, clinical state, and health-related measures at each visit. Following 3 months of treatment, MMJ patients demonstrated improved task performance accompanied by changes in brain activation patterns within the cingulate cortex and frontal regions. Interestingly, after MMJ treatment, brain activation patterns appeared more similar to those exhibited by healthy controls from previous studies than at pre-treatment, suggestive of a potential normalization of brain function relative to baseline. These findings suggest that MMJ use may result in different effects relative to recreational marijuana (MJ) use, as recreational consumers have been shown to exhibit decrements in task performance accompanied by altered brain activation. Moreover, patients in the current study also reported improvements in clinical state and health-related measures as well as notable decreases in prescription medication use, particularly opioids and benzodiapezines after 3 months of treatment. Further research is needed to clarify the specific neurobiologic impact, clinical efficacy, and unique effects of MMJ for a range of indications and how it compares to recreational MJ use.

## Introduction

Currently, 30 states and the District of Columbia have medical marijuana (MMJ) programs or pending MMJ legislation, an additional 16 states have passed laws to allow limited access to MMJ, and an estimated 2.6 million individuals in the United States are certified for MMJ use (Procon.org). Since societal attitudes toward marijuana (MJ) have generally warmed, an increasing number of individuals are turning to MMJ to help treat a variety of medical conditions, as patients often do not achieve full symptom alleviation with conventional medications and experience unwanted side effects. Data gathered from several US surveys of MMJ patients indicate that the most common indications for MMJ use included pain-related concerns (i.e., chronic pain, headaches), psychiatric disorders (i.e., anxiety, depression), and insomnia (Nunberg et al., 2011; Reinarman et al., 2011; Bonn-Miller et al., 2014; Park and Wu, 2017). Although considerable research efforts have clarified the impact of recreational MJ use, particularly among adolescent and young adult populations, to date, there is a paucity of research focused on examining the impact of MMJ use on neurobiologic measures, including brain function and structure. As the number of MMJ patients continues to grow, research efforts designed to understand potential changes associated with MMJ use are critically important.

A large body of evidence from the past several decades suggests that recreational MJ use is related to cognitive decrements, including deficits in verbal memory (Tait et al., 2011; Auer et al., 2016; Shuster et al., 2016), processing speed (Fried et al., 2005; Lisdahl and Price, 2012; Jacobus et al., 2015), attention (Ehrenreich et al., 1999; Cousijn et al., 2013; Becker et al., 2014) and executive function (Crean et al., 2011; Gruber et al., 2012b; Solowij et al., 2012; Dougherty et al., 2013; Hanson et al., 2014; Jacobus et al., 2015; Dahlgren et al., 2016). While these deficits have been observed in adult MJ users (Nader and Sanchez, 2017), they are most salient among MJ-using adolescents (Lisdahl et al., 2014) who are in the midst of critical neurodevelopment (Giedd et al., 1999). Furthermore, these decrements have also been linked to alterations in brain structure and function. Although the directionality of structural alterations appears to be dependent on the brain region under investigation (Batalla et al., 2013), studies show that gray and white matter alterations are associated with increased executive dysfunction (Medina et al., 2009, 2010; Churchwell et al., 2010; Clark et al., 2012; Price et al., 2015). In addition, functional magnetic resonance imaging (fMRI) studies have reported altered activation patterns within the prefrontal cortex as well as orbitofrontal, cingulate, and subcortical/limbic regions of recreational MJ users compared to non-using control subjects during tasks of executive function (Lisdahl et al., 2014, for review). Further, similar to studies of cognitive performance and brain structure, fMRI studies have revealed that earlier onset of MJ use is related to altered patterns of brain activation during tasks requiring cognitive control and inhibition (Tapert et al., 2007; Gruber et al., 2012a; Sagar et al., 2015).

Although many have posited that MMJ use would be associated with similar deficits, preliminary studies have suggested that this may not be the case. In our own recent pilot investigation (Gruber et al., 2016), the only study to date to examine the impact of whole plant-derived MMJ products on cognitive performance, we found that MMJ patients did not demonstrate decrements in performance on measures of executive function following 3 months of MMJ treatment. In fact, patients generally demonstrated *improved* performance on a number of measures, particularly those assessing executive function. Improvements were also noted on several measures of quality of life, sleep, and depression relative to pre-MMJ treatment levels. Differences between recreational and MMJ users may be related to a variety of factors, including age of onset of MJ use, duration, magnitude and frequency of use, and choice of actual cannabis products used. Although products used by recreational MJ consumers and MMJ patients are derived from the same plant species, they are generally utilized for different purposes (i.e., to get high/alter one’s current state of being vs. symptom alleviation). Accordingly, recreational and medical users often seek different MJ products with various constituent compositions based on the desired effect. Recreational MJ users often seek products high in Δ^9^-tetrahydrocannabinol (THC), the main psychoactive constituent of the cannabis plant, and while medical patients may also choose products with high THC levels they often seek products high in other potentially therapeutic cannabinoids. Research has begun to focus on the beneficial effects of cannabidiol (CBD), the primary non-intoxicating constituent of MJ, which has been touted for its antipsychotic, anxiolytic, anti-seizure, and anti-inflammatory properties (Rong et al., 2017). While studies from recreational MJ users have reported a relationship between higher levels of THC and poorer cognitive performance (Ramaekers et al., 2006; Kowal et al., 2015) the acute administration of CBD prior to THC has been shown to *improve* cognitive function (Morgan et al., 2010; Englund et al., 2013), underscoring the need for further study. Moreover, Yücel et al. (2016) recently found that although MJ users exposed to THC exhibit alterations in hippocampal volume and neurochemistry, those who utilized CBD-containing products did not demonstrate differences relative to healthy controls. Similarly, a recent review of the effects of THC and CBD on neuroanatomy concluded that MJ users are prone to brain alterations in regions with high cannabinoid receptor density, and although THC exacerbates these alterations, CBD appears to protect against these deleterious changes (Lorenzetti et al., 2016). In addition, several researchers have administered pure THC or CBD to healthy control participants to investigate the impact of these constituents on brain activation patterns using fMRI. In general, studies suggest that THC and CBD have opposite effects on cognition-related brain activation (Bhattacharyya et al., 2010, 2015; Winton-Brown et al., 2011). This may be related to the fact that THC is a CB1 agonist with strong binding affinity for CB1 receptors, while CBD appears to exert effects through more indirect mechanisms, which include additional receptor types (Zuardi, 2008; Ashton and Moore, 2011). Despite preliminary work investigating the acute effects of pure THC and CBD on neural networks associated with cognitive domains impacted by MJ use, to our knowledge, no studies thus far have examined the impact of treatment with whole-plant-derived MMJ products on brain activation patterns.

In order to investigate whether pilot observations of improved executive function (Gruber et al., 2016) persist with larger sample sizes and to determine whether these changes co-occur with altered brain activation patterns, MMJ patients from an ongoing longitudinal study underwent fMRI while completing the Multi-Source Interference Test (MSIT). The MSIT is a robust measure of cognitive interference, a core facet of executive functioning, which is related to attentional control and inhibitory processing and requires actively shifting attention by inhibiting automatic responses (Lezak et al., 2004). This task reliably activates frontal brain regions associated with executive functioning, particularly the cingulo-frontal-parietal (CFP) network (Bush and Shin, 2006). Given our previous findings (Gruber et al., 2016), we hypothesized that following 3 months of treatment, MMJ patients would demonstrate improved task performance, and that this improvement would coincide with changes in brain activation patterns measured by fMRI. We have previously utilized the MSIT to better characterize patterns of cingulate and frontal brain activation within clinical and non-clinical cohorts (Gruber et al., 2012a, 2017), and although no studies thus far have examined MMJ patients using neuroimaging techniques, we hypothesized that improved MSIT task performance would be associated with increased activation in these regions following 3 months of MMJ treatment. We also posited that these changes would occur in the context of improved mood and quality of life ratings.

## Materials and Methods

### Participants

To date, of 45 consented participants, 41 MMJ patients were enrolled and data from 22 patients’ pre-treatment (Visit 1) and 3-month check-in visits (Visit 2) were available for analyses. In addition, patients who were free of MRI contraindications also completed neuroimaging procedures (*n* = 15). In order to qualify for study entry, patients had to be over the age of 18, and have an estimated IQ of 75 or higher as assessed by the Wechsler Abbreviated Scale of Intelligence (WASI; Wechsler, 1999). In order to minimize the effects of previous MJ exposure on study findings, patients were required to be MJ naïve or be abstinent from MJ use for at least 2 years for their pre-treatment visit. Patients were also required to be certified for MMJ use, or describe a plan to use industrial hemp derived products (which do not currently require certification). All subjects received payment for each study visit, and those who completed MRI procedures were compensated additionally in accordance with Partners IRB-approved protocol procedures.

### Study Design

Prior to participation, all study procedures were explained, and each participant was required to provide written informed consent in accordance with the Declaration of Helsinki. This document and all study procedures were approved by the Partners Institutional Review Board. Eligible participants were enrolled in a larger longitudinal study designed to assess the impact of MMJ on cognition and brain function over the course of 12–24 months. Patients completed all assessments and imaging *prior* to initiation of MMJ treatment and again after 3 months of treatment.

**FIGURE 1:**
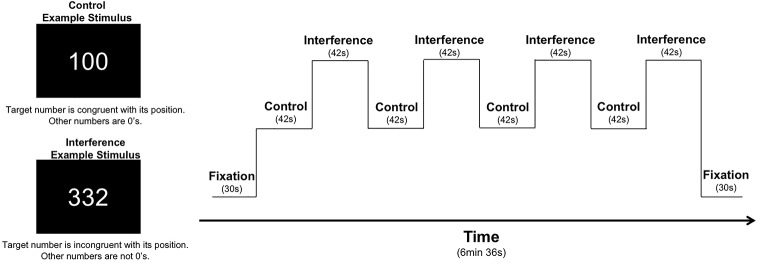
MSIT task design: the MSIT begins and ends with a 30 s fixation. It contains eight total blocks, which alternate between Control (C) and Interference (I) conditions: C, I, C, I, C, I, C, I. Each block is 42 s long, containing 24 trials each, for a total of 384 total trails across the task. For each trial, stimuli are presented for 1.25 s with an interstimulus interval of 0.5 s, which yields a total run time of 6 min and 36 s.

As part of a larger neuroimaging protocol, participants completed the MSIT (Bush et al., 2003; Bush and Shin, 2006) with concurrent fMRI scanning using identical task parameters as reported in our previous studies of recreational MJ users, patients with bipolar disorder, and healthy controls (Gruber et al., 2012a, 2017). Using aspects from well-established measures of cognitive interference (e.g., Stroop, Simon, and Flanker tasks), the MSIT incorporates both spatial and flanker types of interference to measure cognitive control (Bush et al., 2003; Bush and Shin, 2006). During the task, three-digit stimuli sets comprised of the numbers 0, 1, 2, or 3 are presented briefly on a screen. Each set contains two identical distractor numbers and a target number that differs from the distractors. Using a button box, participants report the *identity* of the target number that differs from the two distractor numbers during two conditions: during the Control condition, distractor numbers are always zeros, and the identity of the target number always corresponds to its position on the button box (i.e., 100, 020, 003). During the Interference condition, patients are required to inhibit a prepotent response in favor of a less automatic response (i.e., indicate the identity of the target number rather its position). Distractor numbers are always numbers other than 0, and the identity of the target number is always incongruent with its position on the button box (e.g., 211, 232, 331, etc.). Performance is measured by reaction time and percent accuracy, which can be further subdivided by error type. Omission errors occur when no response is given and are typically reflective of slower or overloaded cognitive processing while commission errors, or incorrect responses, generally indicate difficulty inhibiting inappropriate responses. The entire task is comprised of four blocks of control trials alternating with four blocks of interference trials; the task begins and ends with a fixation period (30 s), making the total run time 6 min and 36 s (see **Figure 1** for graphic representation of the task design).

Patients also completed a battery of self-report rating scales. Briefly, these included the Profile of Mood States (POMS), Beck Depression Inventory (BDI), Beck Anxiety Inventory (BAI), Pittsburgh Sleep Quality Index (PSQI), Barratt Impulsiveness Scale (BIS-11), and the Short Form-36 Health Survey (SF-36), a measure of functional health and quality of life. During each study visit, participants also provided information regarding dose, frequency, and duration of use for all conventional medications, which were categorized into different classes, including opioids, antidepressants, mood stabilizers, and benzodiazepines. Percent change data was calculated to assess potential changes in medication use from pre- to post-3 months of MMJ treatment.

After completing pre-treatment assessments, patients began MMJ treatment at their discretion. Although patients selected their own products and determined their own treatment regimens, we collected detailed data about MMJ use patterns and products. Between study visits, patients submitted biweekly diaries documenting MMJ use and were contacted by phone on a monthly basis to acquire information regarding MMJ product type, frequency, magnitude, and modes of use using a modified timeline follow-back procedure (TLFB; Sobell et al., 1998). Following a minimum of 3 months of regular MMJ treatment, patients returned for their first of several check in visits (Visit 2) where they repeated all study measures. In addition, participants were asked to provide a sample of their most frequently used MMJ product(s) to an outside laboratory (ProVerde Laboratories, Inc.) for cannabinoid constituent profiling. These analyses, which quantified the levels of 10 major cannabinoids including THC and CBD, will be used to identify the unique effects of specific cannabinoids in future analyses.

### Statistical Analyses

Descriptive statistics were calculated for demographic and MMJ use variables. Repeated-measure analyses of variance (ANOVAs) were used to assess changes in clinical state from Visit 1 to Visit 2. The assumption of homogeneity of variance was confirmed using Levene’s F; however, Shapiro–Wilk tests indicated that data for the MSIT were not normally distributed. Accordingly, non-parametric, repeated-measures Wilcoxon Signed Rank Tests were used to assess changes from Visit 1 to Visit 2 for MSIT data. It is of note, however, that the non-parametric tests resulted in similar findings as the ANOVAs; all significant results remained. For the MSIT analyses, alpha was set at 0.05 for the response time and percent accuracy variables. In cases where percent accuracy differed significantly between Visits 1 and 2, comparisons of the two different error types (omission, commission) utilized a Bonferroni correction for multiple comparisons (α/2 = 0.025).

### fMRI Methods and Analyses

All imaging was performed on a Siemens Trio whole body 3T MRI scanner (Siemens Corporation, Erlangen, Germany) using a 12-channel phased array head coil. For the MSIT, 40 contiguous coronal slices were acquired from each participant, ensuring whole brain coverage (5 mm thick, 0 mm skip), and images were collected with TR = 3000, using a single shot, gradient pulse echo sequence (TE = 30 ms, flip angle = 90, with a 20 cm field of view and a 64 × 64 acquisition matrix; in plane resolution 3.125 mm × 3.125 mm × 3.125 mm). A total of 132 images per slice were collected.

fMRI images were analyzed using SPM8 (version 4667, Wellcome Department of Imaging Neuroscience, University College, London, United Kingdom) software package running in MATLAB (version R2010b, MathWorks, Natick, MA, United States). First, blood-oxygen-level dependent (BOLD) fMRI data were corrected for slice timing and for motion in SPM8 using a two-step intra-run realignment algorithm that uses the mean image created after the first realignment as a reference. A criterion of 3 mm of head motion in any direction was used as an exclusionary criterion. The realigned images were then normalized to an EPI template in Montreal Neurological Institute stereotactic space using DARTEL. Normalized images were re-sampled into 3 mm^3^ voxels and then spatially smoothed using an isotropic Gaussian kernel with 6 mm full width at half maximum. Global scaling was not used, high-pass temporal filtering with a cut-off of 168 s was applied, and serial autocorrelations were modeled with an AR(1) model in SPM8. Using a general linear model, statistical parametric images were calculated individually for each subject showing Interference > Control. These images were subsequently entered into second level model, subjected to a voxel-wise contrast and *t*-test to assess statistical significance. In addition to the realignment during the preprocessing, effects of motion were further corrected by removing motion related components from the data by including the calculated motion parameters from the realignment as regressors in the GLM (e.g., six nuisance regressors corresponding to three directions of translation and three axes of rotation). Regions of interest (ROI) masks were created using the Wake Forest University Pickatlas utility (Maldjian et al., 2003) and included cingulate and frontal regions. Specifically, the cingulate ROI was comprised of both bilateral anterior and mid cingulate regions (22,302 voxels) while the frontal ROI was comprised of bilateral superior frontal, middle frontal and inferior frontal gyri (6,857 voxels; see Supplementary Figure 1). These regions were selected as the cingulate (CC) and frontal cortices are associated with inhibitory processing and are reliably activated during the completion of the MSIT (Bush and Shin, 2006; Gruber et al., 2012a, 2017). Contrast analyses consisted of the subtraction of one map from the other; for example, the cingulate activity of Visit 1 was subtracted from cingulate activity of Visit 2 to determine which areas showed increased activity over the course of treatment. As in previous studies (Heckers et al., 2004; Harrison et al., 2007; Yucel et al., 2007; Shin et al., 2011; Harding et al., 2012), the fixation point was not included in planned contrast analyses. The statistical threshold was set at *p* < 0.05 for cluster level family-wise-error (FWE), *p* < 0.001 for voxel level FWE with a minimum cluster extent *k* = 15 contiguous voxels in accordance with previously published manuscripts that have utilized the MSIT and have used a *k*-value of 15 (Gruber et al., 2012a) or lower (Harding et al., 2012; Bush et al., 2013). In addition to utilizing previously published *k*-values, we also conducted Monte Carlo simulations (Ward, 2000) to determine a more rigorous cluster extent for *p* < 0.001 which yielded *k* = 91. As two ROIs were used for analyses, data was also corrected for multiple comparisons, generating a new statistical threshold (*p* < 0.0005). One patient was excluded from MSIT analyses as they requested early termination of scanning procedures.

## Results

### Demographics and MMJ Use

All patients (11 male, 11 female) were between the ages of 28–74 (*M* = 50.64, *SD* = 13.15) who reported seeking MMJ treatment for a variety of conditions including pain (*n* = 13), anxiety/PTSD (*n* = 10), sleep (*n* = 10), mood (*n* = 8), and “other” conditions (*n* = 8), which included gastrointestinal issues, difficulty with attention, and additional indications not specified by the state of Massachusetts. Patients in the current sample were generally well-educated; all had earned a high school diploma, many completed advanced education (*M* = 15.91 years, *SD* = 1.97), and all were of at least average intelligence as measured by the WASI (*M* = 117.23, *SD* = 7.63). Upon initiation of MMJ treatment, all patients reported at least weekly use, which ranged from 1.5 times per week to multiple times per day. As noted in **Table 1**, patients reported using MMJ products an average of 5.34 days per week and 1.83 times per day for an overall average of 10.26 total episodes of MMJ use per week. Patients also indicated various routes of administration, including smoking and vaporizing flower, as well as use of oil and concentrates (vaporized and oral administration), tinctures, edibles, and topicals.

**Table 1:** Demographics and MMJ use.

Demographic variable (*n* = 22)	Mean (*SD*)
Age	50.64 (*13.15*)
Education (years)	15.91 (*1.93*)
WASI^a^ Full Scale IQ	117.23 (*7.63*)

**MMJ use^b^**	

Days of MMJ use/week	5.34 (*1.99*)
Times/day used	1.83 (*1.02*)
Total MMJ use episodes/week	10.26 (*7.71*)

**Mode of use**	**Number of patients**

Smoke (flower)	8
Vaporize (flower)	9
Vaporize (oil/concentrates)	6
Oil/concentrates (non-smoked/vaporized)	5
Tincture	6
Edibles	7
Topicals	2

*^a^WASI, Wechsler Abbreviated Scale of Intelligence; ^b^reflects average use from the start of regular treatment through Visit 2.*

### MSIT Behavioral Performance

Relative to pre-treatment, patients demonstrated improved MSIT performance following 3 months of MMJ treatment (**Table 2**). During the Control condition, patients exhibited improved performance, marked by fewer omission errors; however, qualitative analyses revealed that patients approached near perfect levels of performance pre-and post-treatment for this condition. During the Interference condition, patients performed notably better at Visit 2, demonstrating significantly fewer omission and a trend for fewer commission errors, and thus significantly improved percent accuracy. In addition, MMJ patients also demonstrated faster response times during Visit 2, relative to Visit 1, across both Control and Interference trials.

**Table 2:** Repeated measures Wilcoxon signed rank tests assessing Multi-Source Interference Test (MSIT) performance at pre-treatment and after 3 months of MMJ use (post-treatment).

MSIT variable	Visit 1 Pre-treatment Mean (*SD*)	Visit 2 Post-treatment Mean (*SD*)	Wilcoxon	
			*Z*	*p* (*r*)
**Control condition**				
Response time (ms)	608.90 (*97.20*)	582.62 (*64.97*)	2.062	**0.020 (0.500)^∗^**
Percent accuracy	97.40 (*2.57*)	98.82 (*1.74*)	2.282	**0.011 (0.553)^∗^**
Omission errors^a^	1.73 (*2.25*)	0.68 (*1.09*)	1.974	**0.024 (0.479)^∗^**
Commission errors^a^	0.77 (*0.97*)	0.46 (*0.86*)	1.461	0.072 (0.354)
**Interference condition**				
Response time (ms)	914.23 (*76.56*)	886.62 (*82.76*)	2.743	**0.003 (0.665)^∗^**
Percent accuracy	79.03 (*18.87*)	86.55 (*11.88*)	2.858	**0.002 (0.693)^∗^**
Omission errors^a^	11.96 (*12.01*)	7.27 (*7.92*)	2.750	**0.003 (0.667)^∗^**
Commission errors^a^	8.18 (*9.11*)	5.77 (*5.57*)	1.718	*0.043* (*0.417*)ˆ

*df = 1,21; ^a^corrected for multiple comparisons using Bonferroni method; ^∗^results significant at p ≤ 0.05 when α = 0.05 or, for Bonferroni corrected analyses, at p ≤ 0.025 when α = 0.025; ˆresults trending toward significance at p ≤ 0.10 when α = 0.05 or, for Bonferroni corrected analyses, at *p* ≤ 0.05 when α = 0.025.*

### MSIT fMRI Data

**FIGURE 2:**
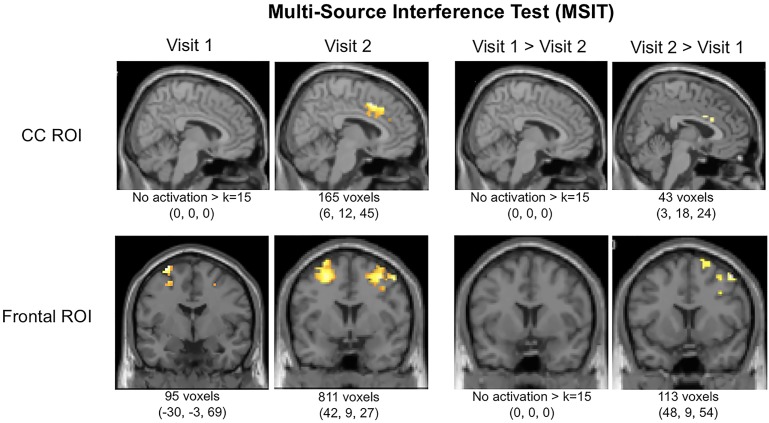
Functional magnetic resonance imaging (fMRI) activation in cingulate cortex (CC) and frontal regions of interest (ROIs) during the MSIT (Interference-Control). Local maxima and total k (voxels activated within ROIs per contrast) are displayed below images.

Interestingly, in addition to improved task performance, MMJ patients exhibited notable changes in brain activation patterns in terms of both magnitude and location from Visit 1 to Visit 2. Results are provided in **Table 3** which includes data for both the *a priori* threshold of *k* = 15 and also indicates which values survived the new threshold of *k* = 91 determined by the Monte Carlo simulations. After initiating MMJ treatment, patients generally exhibited increased activation within both the cingulate and frontal ROIs. Specifically, within the CC ROI, single-sample analyses revealed no significant activation at Visit 1, yet at Visit 2 patients exhibited focal activation within the midcingulate cortex (*k* = 165). Within-subjects contrast analyses between Visit 1 and Visit 2 revealed no significant activation differences for Visit 1 > Visit 2, but the Visit 2 > Visit 1 contrast indicated activation differences within the right anterior cingulate (*k* = 43). Within the frontal ROI, single-sample analyses revealed activation at Visit 1 within the left superior (*k* = 65) and the right inferior frontal gyrus (*k* = 19), and at Visit 2, within the right inferior (*k* = 575), left middle frontal gyrus (*k* = 217), and the left precentral gyrus (*k* = 19). Within-subjects contrast analyses between Visit 1 and Visit 2 yielded no significant activation differences for the Visit 1 > Visit 2 contrast; however, the Visit 2 > Visit 1 contrast revealed significant activation differences within the right middle gyrus (*k* = 88) and superior frontal gyrus (*k* = 25). See **Figure 2**.

**Table 3:** Multi-Source Interference Task (Interference-Control condition): activation local maxima within cingulate cortex (CC) and frontal cortex regions of interest (ROIs).

*ROI* Visit Region	Cluster size (voxels)	*x*	*y*	*z*	SPM {t}	Voxel *p* (FWE - corrected)
***CC***						
**Visit 1**						
No activation *k* ≥ 15	–	–	–	–	–	–
**Visit 2**						
Right middle cingulate cortex	**150**	6	12	45	8.45	<0.0005
Right anterior cingulate cortex	15	12	36	15	5.18	<0.0005
**Visit 1 > Visit 2**						
No activation *k* ≥ 15	–	–	–	–	–	–
**Visit 2 > Visit 1**						
Right anterior cingulate cortex	20	3	18	24	5.41	<0.0005
Right anterior cingulate cortex	23	9	30	18	5.21	<0.0005
***FRONTAL***						
**Visit 1**						
Left superior frontal gyrus	65	-30	-3	69	9.51	<0.0005
Right inferior frontal gyrus (*p. triangularis*)	19	45	12	24	8.48	<0.0005
**Visit 2**						
Right inferior frontal gyrus (*p. opercularis*)	**575**	42	9	27	10.75	<0.0005
Left middle frontal gyrus	**217**	-24	-6	51	10.08	<0.0005
Left precentral gyrus	19	-45	9	36	7.87	<0.0005
**Visit 1 > Visit 2**						
No activation *k* ≥ 15	–	–	–	–	–	–
**Visit 2 > Visit 1**						
Right middle frontal gyrus	88	48	9	54	6.94	<0.0005
Right superior frontal gyrus	25	24	12	66	6.58	<0.0005

*The statistical threshold was initially set at p < 0.05 for cluster level family-wise-error (FWE), and p < 0.0005 for voxel level FWE (corrected for multiple comparisons) with a minimum cluster extent k = 15 contiguous voxels. Bolded results indicate values that survived the Monte Carlo simulation minimum cluster extent (k = 91).*

### Clinical Ratings and Conventional Medication Use

Following 3 months of MMJ treatment, patients reported some improvement on measures of mood and quality of life (**Table 4**). Across all rating scales, no significant worsening of clinical state or quality of life was observed. Moreover, consistent with a previous report (Gruber et al., 2016), patients reported significant *improvements* on measures of depression (BDI), impulsivity (BIS-11), sleep (PSQI), and quality of life (SF-36). Specifically, on the SF-36, patients indicated significantly improved energy/fatigue and fewer role limitations due to physical health, which reflects how often patients’ physical health affects their work and other life activities. A trend also emerged suggesting improved social functioning.

**Table 4:** Mood and health ratings at pre-treatment and after 3 months of MMJ use (post-treatment).

Rating scale	Visit 1 Pre-treatment Mean (*SD*)	Visit 2 Post-treatment Mean (*SD*)	ANOVA	
			*F*	*p* (η^2^)
**Clinical ratings**				
*Profile of Mood States (POMS)*^a^				
Vigor	16.86 (*6.19*)	16.14 (*6.53*)	0.543	0.235 (0.025)
Anger	8.68 (*10.33*)	8.59 (*9.21*)	0.003	0.477 (<0.001)
Confusion	8.00 (*6.38*)	6.73 (*4.92*)	*2.748*	*0.056* (*0.116)ˆ*
Tension	12.59 (*9.96*)	12.05 (*9.97*)	0.153	0.350 (0.007)
Fatigue	9.91 (*7.29*)	8.77 (*7.24*)	*1.946*	*0.089* (*0.085)ˆ*
Depression	13.18 (*16.78)*)	14.14 (*16.95*)	0.217	0.323 (0.010)
TMD	35.50 (*51.08*)	34.14 (*48.89*)	0.055	0.409 (0.003)
*Beck Depression Inventory (BDI)*^a^				
Total	13.77 (*12.60*)	9.73 (*11.65*)	***9.559***	***0.003* (*0.313*)^∗^**
*Beck Anxiety Inventory (BAI)*^a^				
Total	10.55 (*10.32*)	9.73 (*9.93*)	0.227	0.319 (0.011)
**Impulsivity**				
*Barratt Impulsiveness Scale (BIS-11)*^a^				
Attention	16.59 (*5.77*)	16.59 (*5.47*)	0.000	0.500 (0.000)
Motor	23.00 (*5.43*)	21.23 (*5.37*)	***12.531***	***0.001* (*0.374*)^∗^**
Non-planning	23.41 (*5.53*)	23.59 (*5.50*)	0.077	0.392 (0.004)
Total	63.00 (*15.08*)	61.41 (*14.64*)	1.626	0.108 (0.072)
**Health and quality of life ratings**				
*Pittsburgh Sleep Quality Index (PSQI)*^b^				
Total	8.26 (*4.46*)	6.05 (*3.26*)	***7.167***	***0.008* (*0.285*)^∗^**
*SF-36*^a^				
Physical functioning	71.59 (*21.40*)	71.82 (*26.03*)	0.002	0.483 (<0.001)
Role limitations (physical)	44.32 (*43.60*)	56.82 (*44.44*)	***3.915***	***0.031* (*0.157*)^∗^**
Role limitations (emotional)	63.64 (*43.53*)	60.61 (*45.58*)	0.096	0.380 (0.005)
Energy/fatigue	42.73 (*25.39*)	51.36 (*21.67*)	***10.738***	***0.002* (*0.338*)^∗^**
Emotional well-being	67.64 (*26.18*)	65.82 (*27.32*)	0.827	0.187 (0.038)
Social functioning	62.50 (*29.12*)	68.75 (*29.06*)	*1.819*	*0.096* (*0.080*)ˆ
Pain	52.73 (*2.05*)	56.59 (*26.06*)	0.918	0.174 (0.042)
General health	57.50 (*19.75*)	60.91 (*19.56*)	1.668	0.105 (0.074)

*Clinical rating scales (POMS, BDI, BAI), lower scores reflect lower levels of clinical symptoms; BIS-11, lower scores indicate lower levels of self-reported impulsivity; PSQI, lower scores reflect improved sleep quality; SF-36, higher scores indicate higher quality of life. ^a^df = 1,21; ^b^df = 1,18; ^∗^bolded results are significant at p ≤ 0.05; ˆ italicized results approach significance at p ≤ 0.10.*

In addition to improvements in clinical state and quality of life, following 3 months of MMJ treatment, patients reported reductions in their use of conventional pharmaceutical products across several drug classes. Specifically, patients taking opioids reported a 47.69% reduction in use and those prescribed benzodiazepines reported a 46.91% reduction in use. Antidepressant use decreased by 22.35% while the use of mood stabilizers decreased by 28.57% between Visit 1 and Visit 2.

## Discussion

Following 3 months of MMJ treatment, patients exhibited improved task performance and related alterations in frontal brain activation patterns during the completion of the MSIT, a measure of executive function and cognitive control, relative to pre-MMJ treatment. Within the cingulate cortex (CC), patients did not exhibit any significant pre-treatment activation during the Interference condition of the MSIT; however, after 3 months of treatment, robust activation was noted within this region. In fact, the magnitude of activation significantly increased over the course of treatment such that post-treatment activation patterns appeared more similar to that of healthy controls observed in previous studies (Bush and Shin, 2006; Gruber et al., 2012a). Activation within the frontal ROI was also notably increased following 3 months of MMJ treatment relative to pre-MMJ treatment. Taken together, these changes may be reflective of a potential “normalization” of brain function following 3 months of MMJ use.

Further, changes in brain activation patterns were observed in the context of improved task performance and self-reported improvements in mood and quality of life as well as reduced sleep disturbance and lower motor impulsivity, consistent with previously published preliminary data (Gruber et al., 2016). It is possible that improvements in symptomatology (i.e., relief of symptoms, improved mood/sleep) are directly related to observed improvements in cognitive function and alterations in brain activation. Patients in the present study most commonly endorsed pain and anxiety as their reasons for MMJ certification; both of these conditions have previously been associated with reduced cognitive performance (Moriarty et al., 2011; Vytal et al., 2013). Symptom improvement may therefore result in improved cognitive performance, and subsequently impact patterns of brain activation during completion of these tasks.

In addition to reduced symptomatology resulting in improved cognitive performance, it is also possible that several other factors may have also contributed to the observed changes. Patients reported notable decreases in their use of opioids, benzodiazepines, antidepressants, and mood stabilizers, and it is possible that reductions in conventional medications influenced changes in brain activation patterns. In fact, several studies have shown that mood stabilizers, benzodiazepines, and antidepressants generally attenuate activation (Bell et al., 2005; Del-Ben et al., 2005; Paulus et al., 2005; Arce et al., 2008; Murphy et al., 2009). In particular, Bell et al. (2005) reported that use of mood stabilizers is related to hypoactivation of the CFP network, a key region implicated in cognitive interference processing. While we are not aware of fMRI research focused on the effects of short-term prescriptive doses of opioids in humans, one study examining opioid dependence reported that normalization of frontal brain activation patterns was related to days since last drug use (Bunce et al., 2015). Reduction or cessation of use of these medications may therefore alter patterns of brain activation. Accordingly, future studies are needed to disentangle the effects of MMJ treatment and conventional medication use on brain activation patterns, and may benefit from limiting clinical samples to only those on a single specific class of conventional medication.

Although findings from this study indicate *improvements* in cognitive task performance and more normalized patterns of brain activation after 3 months of MMJ treatment, previous studies, exclusively focused on recreational MJ users, have reported *decrements* in cognitive performance and accompanying atypical neural alterations. A recent review highlighting neuroimaging findings in recreational MJ users found evidence for altered frontal neural function during completion of executive function tasks (Weinstein et al., 2016), a finding observed in our own previous research (e.g., Gruber and Yurgelun-Todd, 2005; Gruber et al., 2012a; Sagar et al., 2015). A number of critical factors may account for the differences between our current findings in MMJ patients relative to findings from recreational MJ consumers. The majority of studies of recreational MJ use have included adolescent and young adult populations. Overwhelmingly, studies have demonstrated that early/adolescent onset of recreational MJ use is related to poorer task performance and changes in brain structure and function (Lisdahl et al., 2013, 2014; Jacobus and Tapert, 2014; Levine et al., 2017). Given that participants in the current sample are adults (*Mean age* = 50.64) who are well-beyond the critical stages of neurodevelopment (Giedd et al., 1999), they are likely less vulnerable to the adverse neural effects of THC. Interestingly, recent preclinical evidence indicates that THC may have the potential to *improve* cognition in older individuals (Bilkei-Gorzo et al., 2017). Mature and old mice administered low doses of THC demonstrated a reversal of age-related cognitive decline, hypothesized to be related to upregulation of the aging endocannabinoid system via increased signaling secondary to low dose THC exposure. Moreover, the same exposure resulted in cognitive decrements among young mice. Additional research is needed to more fully understand the mechanisms underlying these improvements and to examine the impact of cannabis and cannabinoids in older adult populations as well as the effects of low doses of THC, as these factors likely influence the impact of MJ use.

It is also important to consider patterns of MJ use, including frequency and duration of use, in order to understand potential reasons for the different outcomes among recreational users and medical patients. In the current study, all patients reported using MMJ at least weekly; on average, they reported using 5 days per week and 1–2 times per day. Traditionally, studies of recreational users have examined chronic, heavy use; although criteria for “heavy use” can vary across investigations, most studies have required participants to use MJ at least 1–4 days per week (Tait et al., 2011; Gruber et al., 2012a; Macher and Earleywine, 2012; Dougherty et al., 2013; Cousijn et al., 2014), similar to the frequency of use among the current sample of medical patients. Given these similarities, it is unlikely that differences between recreational and MMJ patients are solely attributable to frequency of use. Additionally, studies of recreational MJ users typically include consumers with a longer duration of MJ use relative to the current sample of MMJ patients, and differences in cumulative exposure should also be considered. For this reason, our ongoing study is designed to examine MMJ users after increasingly longer periods of use to explore the impact of longer durations of MMJ use on cognitive function.

Further, MMJ patients and recreational MJ users also typically differ in terms of the products they use. Recreational MJ products are often prized for high THC levels, and the goal of the recreational consumer is to change their current state of being or to ‘get high.’ MMJ patients seek symptom alleviation and tend to choose products with rich and varied cannabinoid profiles including constituents other than THC, which may also impact clinical state, cognitive processing and other domains. For example, CBD, which has been touted for its clinical benefits (Rong et al., 2017), has demonstrated efficacy in mitigating the negative cognitive effects of THC (Yücel et al., 2016) and appears to exert opposite effects on task-related brain activation relative to THC (Colizzi and Bhattacharyya, 2017). In addition, although there is a paucity of research in this area, some studies have examined the direct impact of acute CBD administration on cognitive performance. Englund et al. (2013) reported that administration of CBD prior to the administration of THC resulted in better episodic memory relative to placebo pre-treatment in healthy controls. Morgan et al. (2010) examined verbal memory performance in current recreational MJ users and found that those using products without CBD (confirmed by hair sample analysis) performed more poorly on verbal memory measures than those with detectable levels of CBD. While no studies have examined the impact of whole plant-derived MMJ products or assessed the long-term impact of MMJ treatment, some studies have utilized fMRI techniques to examine the acute effects of individual cannabinoids. Borgwardt et al. (2008) studied the acute impact of THC, CBD, and placebo on executive function in healthy controls using a Go/No go task. Although the authors did not report any performance differences between cannabinoids or placebo, fMRI data demonstrated that THC reduced activation in frontal and anterior cingulate regions, while CBD reduced activation in temporal and insular regions relative to placebo. In addition, Bhattacharyya et al. (2010) found that intravenous administration of THC and CBD resulted in opposite effects on brain activation patterns across multiple regions during the completion of memory, inhibitory function, and affective measures. Given these findings, data from the present study may reflect the direct neurobiologic effects of cannabinoids, as increased endocannabinoid signaling is associated with improved cognition (Egerton et al., 2006), reduced stress response, emotional regulation, and increased endogenous reward signaling (Hill and McEwen, 2010; Befort, 2015), and as previously noted, specific alterations in brain activation patterns (Borgwardt et al., 2008; Bhattacharyya et al., 2010). Results may also reflect the indirect impact of whole plant-derived products, which include an array of cannabinoid constituents, and may exert downstream affects on multiple receptor types and neural systems (i.e., pain and reward circuitry). While THC and CBD are generally the most abundant cannabinoids in patients’ products, and as noted, several studies have begun to explore their impact on cognition and brain activation patterns, a number of other cannabinoids including cannabigerol (CBG), cannabinol (CBN), cannabichromene (CBC), and tetrahydrocannabidivarin (THCV), are often present in MMJ products, and may have moderated or indirectly affected the impact typically associated with THC exposure (Englund et al., 2016). Further research is clearly indicated for assessing the specific impact of individual cannabinoids on cognitive and clinical variables in patients using cannabis for medical purposes.

### Limitations and Future Directions

Despite the compelling nature of the study findings, several limitations must be noted. First, the current investigation is designed as an observational, longitudinal pre–post study in which patients choose their own products and treatment regimen. The ability to assess the impact of whole plant-derived cannabis-based products is more ecologically valid than studies involving synthetic or non-plant derived products; however, the current legal landscape prohibits the use of dispensary-based products within a clinical trial model and allows only the use of products supplied by the National Institute on Drug Abuse (NIDA). While NIDA’s drug supply program has expanded their portfolio of MJ products available for research, their supply does not currently include the range and scope of products (i.e., product type, potency, constituent profiles, etc.) that patients are seeking and obtaining through dispensaries and caregivers across the nation. Accordingly, as a clinical trial model could not be utilized, the present study collected comprehensive data on product source, selection, dose, frequency, and mode of use. Further, as previously noted, patients also provided a sample of their most frequently used MMJ products for cannabinoid constituent profiling. Interestingly, 13 of the 22 patients (59%) in the current study were identified as taking products high in CBD which may have contributed to study findings given previous data highlighting its clinical benefits (McGuire et al., 2017; Rong et al., 2017). As initial laboratory analyses revealed a range of cannabinoid constituents from patients’ products, additional analyses will be conducted to examine the impact of specific cannabinoids and their relationship with cognitive and clinical variables. Further, future studies will assess potential differences between patients who choose products high in THC compared to those using products high in CBD, as these data may provide critical information regarding efficacy of individual constituents and combinations of constituents for specific indications and conditions to better inform selection of MMJ products.

The current study utilized a pre–post, within-subjects design in which all patients are MJ naïve at Visit 1 and are followed over the course of 12–24 months in order to clarify the impact of prolonged duration of exposure to MMJ treatment. As results from the current study represent only data from baseline and patients’ first check-in visit after 3 months of MMJ treatment, data must be considered preliminary. Additional analyses, which are planned for the future, are needed to understand the impact of MMJ over longer treatment periods. Further, as a result of the pre–post study design, repeated administration of cognitive measures was required, and thus practice effects cannot be completely ruled out. Although no studies to date have specifically examined practice effects for the MSIT, given the lengthy duration of time between visits (at least 3 months) and the computerized nature of the task, it is highly unlikely that practice effects would persist. In addition, given the longitudinal nature of the design, each subject’s baseline assessment serves as their own control from which to assess change after initiation of MMJ treatment. It could, however, prove beneficial for future investigations to also recruit a control group of patients who report similar symptoms (i.e., pain, insomnia, anxiety, etc.) but who do *not* choose to utilize MMJ. Comparing outcomes of MMJ patients and “treatment as usual” patients over time could strengthen findings if MMJ patients display more positive outcomes relative to those who do not use MMJ but suffer from similar symptoms or conditions.

In addition, statistical thresholds for fMRI analyses were set in accordance with previous investigations (Shin et al., 2011; Gruber et al., 2012a; Harding et al., 2012) in order to aid in the interpretation of findings. While more stringent thresholds were also applied and are noted within the results, it is important to recognize that some findings did not survive the more rigorous thresholds, a common issue in fMRI studies with limited sample sizes.

In order to gain a more thorough understanding of how MMJ impacts cognitive functioning, it will be important for future studies to replicate current study findings using other measures of executive function and to examine additional cognitive domains. As executive functioning has been shown to be impacted by recreational MJ use (for review, Crean et al., 2011), this domain was targeted for the current investigation; however, it is crucial to examine additional cognitive variables, including verbal memory, which has also been shown to be sensitive to MJ use (for review, Solowij and Battisti, 2008; Broyd et al., 2016).

Finally, this study included MMJ patients using products for a variety of indications, which resulted in a varied clinical sample. While this approach provides a broad assessment of the impact of MMJ, it is likely that individual conditions and symptoms will have unique patterns of responses associated with MMJ treatment. A number of medical and psychiatric conditions have been shown to negatively impact cognitive processing; accordingly, future studies may derive additional power by limiting inclusion to patients with a single condition or indication (i.e., patients using MMJ exclusively for pain) or including only patients taking medications from a single drug class.

## Conclusion

To our knowledge, this study represents the first neuroimaging investigation of patients using marijuana for medical purposes. Following 3 months of MMJ treatment, brain activation patterns appear more similar to those exhibited by healthy controls from previous studies than at pre-treatment. This finding provides strong evidence that MMJ treatment may normalize brain activity. Importantly, these changes were accompanied by improved task performance as well as positive changes in ratings of clinical state, impulsivity, sleep, and quality of life. Further, patients reported notable decreases in their use of conventional medications, including opioids. In light of the national opioid epidemic, these data clearly underscore the need to expand and extend this study to determine if a reduction in opioid use persists with continued MMJ treatment. Results from the current study raise the possibility that the observed improvements in cognition and related changes in functional activation patterns may be related to direct and/or indirect effects of cannabinoids, specifically within an adult population beyond the stages of critical neuromaturation. Patients utilizing MMJ appear to use products with different cannabinoid profiles (i.e., high CBD) relative to recreational users, which is also likely to impact cognitive function. Observed changes may also be related to secondary or more indirect effects, including the reduction of clinical symptoms, improved sleep, and decreased use of conventional medications. Additional studies using both observational and clinical trial models to examine the impact of actual MMJ products used by patients are needed to clarify the underlying neural mechanisms associated with clinical and behavioral changes that accompany MMJ treatment.

## Author Contributions

SG conceptualized and designed the current study in consultation with SL. KS assisted SG in manuscript preparation with additional help provided by the remaining authors. SG, KS, RS, AL, and KC recruited the patients, carried out the study procedures and administered the neuropsychological and clinical assessments. MD completed the statistical analyses and AG completed the neuroimaging analyses.

## Conflict of Interest Statement

The authors declare that the research was conducted in the absence of any commercial or financial relationships that could be construed as a potential conflict of interest.
