# Supplementary material for: The Grass Might Be Greener: Medical Marijuana Patients Exhibit Altered Brain Activity and Improved Executive Function after 3 Months of Treatment
Source: Front Pharmacol. 2018 Jan 17;8:983. doi: 10.3389/fphar.2017.00983 (PMC5776082; doi:10.3389/fphar.2017.00983)

Supplementary Figure 1. Illustrative image depicting cingulate (blue) and frontal (red) ROI masks which were generated from WFU Pickatlas utility. Panel A illustrates the cingulate ROI and location for panel B (y = 22).

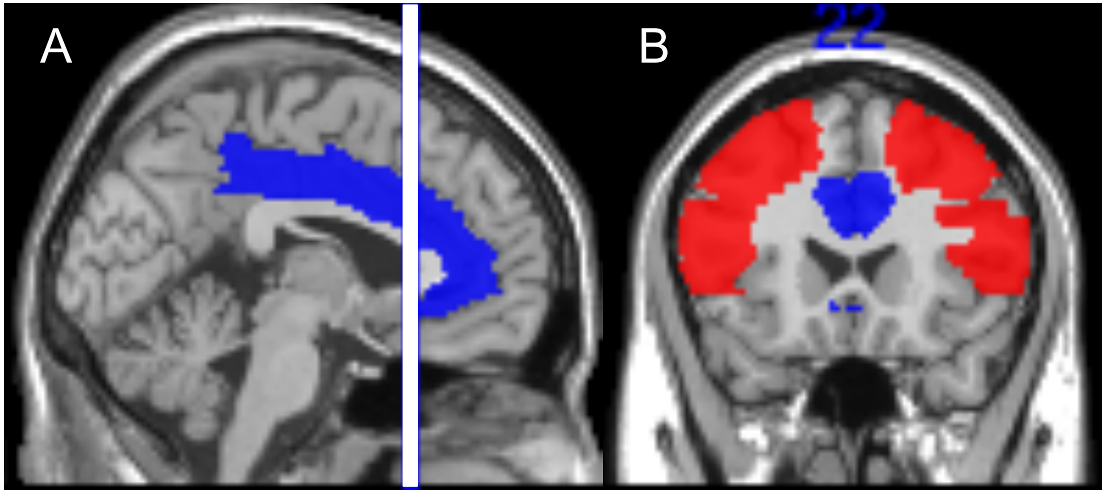

Supplement: Supplementary file 1 [file Image_1.PDF]
